# Retinal Response of Low Myopes during Orthokeratology Treatment

**DOI:** 10.3390/jcm9082649

**Published:** 2020-08-14

**Authors:** António Queirós, Ana F. Pereira-da-Mota, Jéssica Costa, Ana Amorim-de-Sousa, Paulo R. B. Fernandes, José M. González-Méijome

**Affiliations:** Clinical & Experimental Optometry Research Lab (CEORLab), Center of Physics, School of Science, University of Minho, Gualtar, 4710-057 Braga, Portugal; ana_filipa96@hotmail.com (A.F.P.-d.-M.); jeka_tatiana@hotmail.com (J.C.); ana.amorim.sousa@gmail.com (A.A.-d.-S.); pfernandes@fisica.uminho.pt (P.R.B.F.); jgmeijome@fisica.uminho.pt (J.M.G.-M.)

**Keywords:** orthokeratology, myopia control, pattern electroretinography, visual evoked potentials

## Abstract

The aim of this study was to evaluate the changes in retinal activity during orthokeratology (OK) treatment in 20 myopic eyes. Pattern electroretinography (PERG) and visual evoked potential (VEP) were assessed with the RETI-port/scan21 (Roland Consult, Wiesbaden, Germany). Measurements were taken at baseline (BL) and 1 night (1N), 15 nights (15N), 30 nights (30N), and 60 nights (60N) of OK lens wear. Repeated measures analysis of variance (ANOVA) and the Friedman test were used. Twenty eyes (23.20 ± 3.46 years, 70% female) with visual acuity ≤ 0.00 logMAR in post-treatment showed that despite a slight increase in retinal and cortical response amplitude, observed with both PERG and VEP, respectively, immediately after the initial treatment, these differences found were not statistically significant during the 60 days of OK treatment, despite a statistically significant increase in N95 response with PERG. This shows that retinal and cortical visual-related electrical activity is maintained or slightly increased during OK treatment.

## 1. Introduction

Orthokeratology (OK) has been reported to be one of the most effective optical strategies to control myopia progression [1,2] OK lenses reshape the corneal curvature of the eye during sleep, reducing the thickness of the central epithelium and increasing the thickness of the midperipheral area of the cornea [3]. Considering that the cornea is the main optical element of the eye, changes in its shape directly influence the power of the eye, and its focus. The thickening of the midperipheral cornea [4] decreases the hyperopic defocus of the peripheral retina in myopic eyes, which has been hypothesized to slow myopia progression [5]. OK treatment induces significant changes to the corneal topography [6], including an increase in the off-axis focalization properties of the eye [7,8,9], and higher order aberrations [10,11]. It is known that the retina is involved in the development of myopia, and the eye growth is a visually-guided process [12]. Animal studies demonstrate that disruption of normal visual experience may lead to a myopia [13,14].

Retinal function may be assessed by a series of electroretinogram (ERG) tests, including pattern electroretinography (PERG), which provides information about retinal ganglion cell function, and the function of the macula [15]. The PERG is important in clinical and research applications because this test facilitates the interpretation of an abnormal visual evoked potential (VEP), establishing whether the abnormality is present in the retina or along the visual pathway. In both tests, the patterned stimulus is similar. VEPs provide important information regarding the function of the visual pathway, therefore, the response is influenced by the eye, retina, optic nerve, optic radiations and the occipital cortex [16].

Studies on single-flash full-field ERG and multifocal ERG showed reduction in ERG amplitude, and a greater delay of implicit time in human myopic eyes [17,18,19,20]. Several hypotheses have been suggested to explain why ERG changes occur in myopic subjects. First, the reduction in ERG amplitudes may be related to the increased distance from the electrical sources (the retina) and the electrodes as a result of excessive axial elongation. Chen et al. [21] proposed that this reduction can be related to a low retinal cell responsivity. Chan and Mohidin [22] also suggested that an increased axial length of the myopic eye can contribute to the decrease in ERG, due to an increase in subretinal space or a change in the morphology of the retinal cells. Kawabata and Adachi-Usami [17] found that reduced amplitude and delayed latency times in myopic eyes mainly resulted from cone function loss.

There is an increasing interest in understanding the mechanism of development of myopia, to reduce its progression and prevent the development of ocular complications associated with axial elongation such as retinal detachment, macular degeneration, cataracts, and glaucoma [23]. Considering the impact of OK in the optical quality of the eye and the potential involvement of visually guided mechanisms in ocular growth control during OK treatment, the present study was designed to measure the electrical activity in the retina and visual cortex with PERG and VEP, respectively. According to our knowledge, this study is the first one evaluating retinal and visual cortex function in myopic eyes during OK treatment.

## 2. Materials and Methods

### 2.1. Study Design and Subjects

This was a prospective, longitudinal study involving participants fitted with OK lenses in both eyes for overnight wear. Twenty myopic eyes were included after a comprehensive optometric examination. The inclusion criteria included subjects who presented a distance spherical equivalent error from −1.00 to −2.00 D and ≤1.50 D of astigmatism. No previous or current use of contact lenses or contraindications for OK lens wear. The subjects did not suffer from any current eye disease or injury and were not taking any ocular or systemic medication.

Following the recommendations of the Declaration of Helsinki, all subjects received information about the study before they agreed to participate and signed a consent form. The protocol of the study has been reviewed and approved by the Ethics Subcommittee for Life and Health Sciences of the University of Minho. The study was conducted at the Clinical and Experimental Optometry Research Lab (CEORLab), at the University of Minho (Braga, Portugal).

The measurements were taken at baseline (BL) before any lens wear, and they were repeated after 1 (1N), 15 (15N), 30 (30N) and 60 nights (60N) of OK lens wear. Baseline measurements were performed through the distance subjective refraction (spherical equivalent refractive error) with a single vision contact lens (SVCL) measured at the baseline examination. The intraocular pressure was checked with a non-contact tonometer before and after treatment [24].

### 2.2. Contact Lenses

Subjects were fitted with Paragon CRT^®^ 100 LENS (Paragon Vision Sciences, USA) with an optical zone of 5 mm in both eyes for 2 months [25]. OK lenses were fitted by the same practitioner, according to the manufacturer’s recommendation, and the average lens fitting parameters were as follows: (BCR = 8.30 ± 0.25 mm; RZD = 518.75 ± 17.45 μm; LZA = 32.85 ± 0.73°). Subjects were instructed on the use of OK lenses, including instructions on lens insertion and removal, cleaning of lenses, wear regimen, and follow-up visits required. A single-vision silicone-hydrogel SVCL (Biofinity-CooperVision^®^, comfilcon A) was used in baseline measurements, taking into account that with and without the lens the ERG signal would not produce significant changes in the ERG response, as shown in a previous mfERG study [26], which can be an alternative technique to ophthalmic lens in the baseline.

### 2.3. Electrophysiology

**Figure 1:**
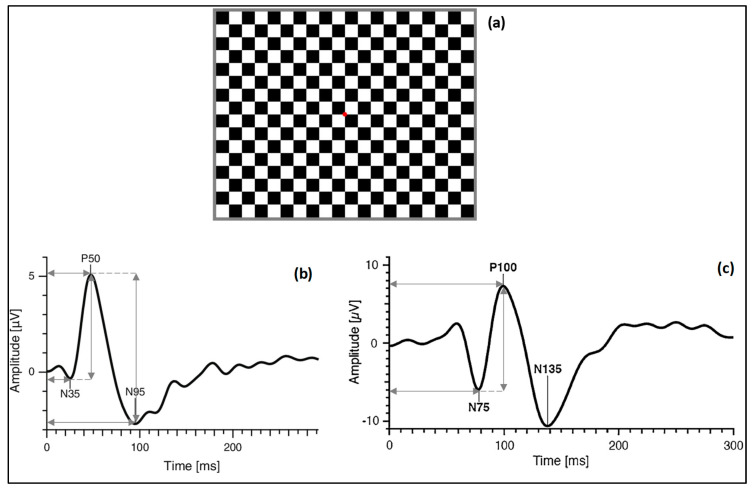
(**a**) Checkerboard pattern with red fixation point; (**b**) typical waveform of pattern electroretinography (PERG), horizontal arrows indicate implicit time of N35, P50 and N95 (ms) and vertical arrows indicate amplitude values of P50 and N95; (**c**) typical waveform of visual evoked potential (VEP), horizontal arrows indicate implicit time of N75 and P100 (ms) and vertical arrow indicates amplitude of P100.

PERG and VEP responses were assessed with the RETI-port/scan21 (Roland Consult, Wiesbaden, Germany) following the International Society for Clinical Electrophysiology of Vision (ISCEV) guidelines [15,16]. The patients were seated comfortably and were instructed to fixate on the center of a pattern-reversal black and white (99% contrast) checkerboard stimuli (Figure 1a), with a check size of 1 degree (0.70 cpd), displayed on a 19-inch RGB computer monitor placed 1 m in front of the eyes, which corresponded to a total angular 48′. The luminance of the white squares was 220.32 ± 1.23 cd/m^2^, and of the black squares was 1.47 ± 0.06 cd/m^2^, which resulted in a calculated Michelson contrast of 98.7%. The mean overall background luminance of the screen was maintained during the measurements (152.64 ± 0.94 LUX). The stimulus was presented at a 4.29 Hz, with a sample frequency of 1.703 kHz and a bandpass filter of 1–50 and 5–50 Hz for VEPs and PERG, respectively.

PERG recordings were performed monocularly in both eyes with the contralateral eye covered with a black patch. In this test, the gold-cup reference and ground electrodes were placed 10 mm lateral to the outer canthus of the tested eye and at the central forehead, respectively. The active electrode used was the Dawson–Trick–Litzkow (DTL), plus an electrode placed onto the lower fornix to make contact with the cornea. VEP recordings were performed in both eyes, monocularly and binocularly. In this case, the gold-cup reference electrode was placed at the central forehead, the ground electrode at the vertex, and the active electrode at the occipital scalp over the visual cortex. The impedance was maintained below 10 kOhm. Fixation was continuously monitored by the technician using a system of in-built camera to minimize the artefacts and ensure patient cooperation. The measurements were done under non-dilated conditions.

From the PERG signal, N35, P50 and N95 wave components were evaluated in terms of amplitude (measured between the troughs and peaks for P50 and N95, in μV) and implicit time (measured from the onset of the stimulus to peak or trough of interest, in milliseconds), see Figure 1b.

Concerning the VEP signal, the implicit time of N75 and P100 were evaluated (measured from the onset of the stimulus to the peak of the component of interest, in milliseconds) and the amplitude of P100 (amplitude between N75 and P100, in μV), see Figure 1c.

### 2.4. Statistical Analysis

Statistical analysis was conducted using SPSS v.24.0 (IBM Co, Armonk, NY, USA). The descriptive data are presented in Mean ± Standard Deviation. The normality of all variables was evaluated using the Shapiro–Wilk test. For paired comparison after and before OK treatment, paired-samples *t*-test was used when normality could be assumed, and the Wilcoxon signed rank test when normality could not be assumed (Table 1). Repeated measures analysis of variance ((ANOVA) for normally distributed variables) and the Friedman test (for non-normally distributed variables) were used to evaluate for potential differences in retinal signal (PERG, Table 2) or cortical signal (VEP, Table 3) of baseline data among four visits with OK treatment (Baseline, 1 Night, 15 Nights, 30 Nights, 60 Nights). Post hoc tests with Bonferroni adjustments were done to correct the level of significance, due to multiple comparisons for different visits (SPSS adjusted). Differences were considered statistically significant when the p-value was less than 0.05.

## 3. Results

### 3.1. Subjects and Study Design

Twenty myopic eyes, with a mean age of 23.20 ± 3.46 years (ranging from 20 to 30), out of which 14 were female, with a mean keratometry horizontal 7.84 × 175° and vertical 7.72 × 85° were included in the study. Table 1 shows the pre-treatment and post-treatment demographic data of the refractive error and visual acuity of the subjects. As we can see in the table, the refractive error, M, after treatment is residual and there are no statistically significant differences for visual acuity before and after the treatment.

### 3.2. Pattern Electroretinography (PERG)

**Figure 2:**
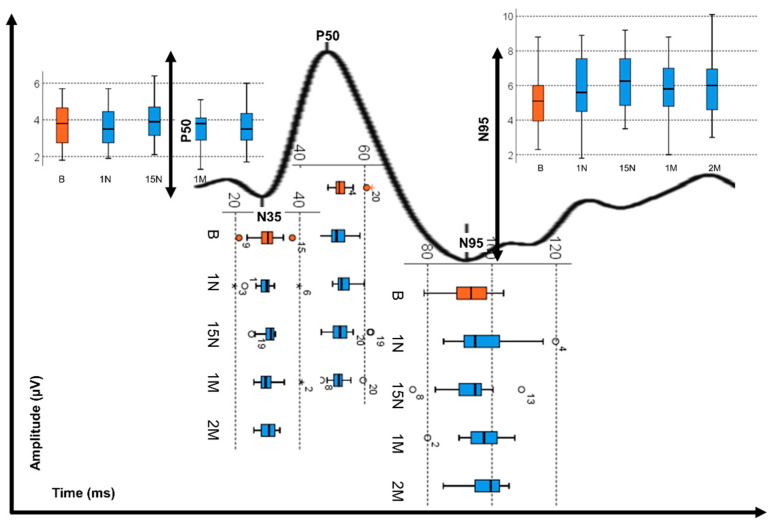
Box plot presentation of the variation of the implicit peak times (N35, P50 and N95) and the variation of the peak amplitude (P50 and N95) of the PERG wave before OK treatment (B-orange) and after OK treatment (Blue: 1N—1 night, 15N—15 nights, 1M—1 month, 2M—2 months). The circles and asterisk refer to outliers found in the measurements corresponding to the number of the eye shown in the figure.

The PERG analysis shows that after the OK treatment, the implicit mean time of N35 does not change significantly, with this verified before the treatment (*p* = 0.649, Friedman test). In contrast, P50 and N95 peaks presented longer response times after OK treatment (*p* < 0.032, Friedman test, Figure 2). As can be seen in Table 2, no significant differences were found for the amplitudes N35_P50 and P50_N95. However, the OK treatment shows that the P50_N95 amplitude values are higher on average by 1 μV after OK treatment.

### 3.3. Visual Evoked Potential (VEP)

The analysis of Table 3 shows that despite the increase in the implicit time of the visual cortex response (N75 and P100) during the treatment with OK, this difference is not statistically significant compared to the response obtained at the beginning of the treatment (*p* > 0.118). The analysis of the VEP also shows similar results for the amplitude P100, although in this case with some oscillations (*p* = 0.093). Thus, we can observe that the treatment of OK throughout 60 nights did not significantly influence the response of visual evoked potentials.

## 4. Discussion

Myopic eyes generally present a relative hyperopic midperipheral retinal defocus [27]. As mentioned, OK is an effective treatment for myopia control, by reshaping the corneal curvature and thickness which produces a myopic shift of the peripheral retinal defocus in myopic eyes, resulting in an emmetropic or slightly myopic periphery while maintaining the central refractive error corrected [5]. The present study shows the changes in the overall electrophysiological response of the ganglion cells of the retina (PERG) and at the visual cortex level (VEPs) over a 60 day period of OK treatment of young subjects with stable low myopia. To our knowledge, this is the very first study evaluating the electrophysiological response of the visual system in subjects who underwent OK treatment for myopia.

Recently, Anders et al. evaluated the effect of 0.01% atropine eye drop application used in myopia prevention over 14 days on the PERG response of 14 young subjects with refractive errors between +2.00 and −2.00 D [28]. They found that atropine did not change considerably the retinal response, which is similar to the present study with OK treatment. However, we have to bear in mind that atropine application and OK are two very different types of treatment to achieve a similar outcome through different pathways. Additionally, Anders et al. used a different PERG methodology, the steady-state PERG, while our recordings rely on standard transient PERG methodology.

Myopes generally have lower electrophysiological amplitudes and delayed responses compared to normal eyes [17,18,19,20]. Other studies induced spherical defocus and found a symmetrical decrease in amplitude and increasing latency of the positive peak (P50) of the PERG and pattern-reversal VEPs (P100) with both myopic and hyperopic defocus [29,30,31,32,33]. The longer axial lengths of myopes were considered the main cause, either by the greater distance from the electrodes to the retina, or by the increase in subretinal space and cellular morphology changes [17,22]. Hidajat et al. evaluated the influence of the axial length of normal eyes on PERG. They found an inverse correlation between the amplitude of ERG and axial length, so that in an average eye larger than 23.8 mm, the P50 amplitude would be 11.6% decreased for each mm of increase in axial length [34]. These results reinforce the possibility of axial length influence on ERG response, and that it should be considered in the analysis of electrophysiological evaluations of the retina. In the present study, the axial length was not measured, so this effect cannot be analyzed, which leads us to consider this a potential limitation of this study. However, the subjects included in the study were low myopes and are expected to not present retinal structural changes as strong as high myopes—this might influence the electrophysiological response. It is not expected that the retinal function in these low myopes might be altered by axial elongation effects.

The results of this study showed that, within a period of two months of OK treatment, the retinal response suffered a small delay at macular (P50) and optic nerve (N95) level, which was not observed in the response of the pattern-reversal VEPs. Some previous studies report different sensitivities between PERG and VEPs in refractive errors, check size [31], pattern contrast [35] and luminance [36]. These studies showed that PERG tend to be more sensitive, associating decreased PERG amplitudes and delayed latencies with the increase in induced defocus, smaller check sizes and decreased contrast and luminance [26,28,29]. OK treatment increases the corneal and total high-order aberrations (HOA) due to the corneal curvature and thickness reshaping. Although the literature describes the increase in the perception of light disturbances in eyes treated with OK lenses associated with a special increase in the spherical-like HOA [37], little is known about the influence of HOA on the electrophysiological response of the visual system. Only recently, Yang et al. evaluated the impact of correcting the HOA of young subjects with an adaptive-optics system in the pattern-reversal VEPs. They observed an increase in the amplitude of the first positive and second negative of the VEP wave for the spatial frequencies from 1 to 16 cpd after correcting HOA, with the biggest amplitude achieved at 1, followed by 16 and 8 cpd. However, these improvements were not as steady as the improvements observed in the CSF (major improvement for higher spatial frequencies, followed by lower). We suggest that the delay in the P50 peak may be associated with the blur induced by the HOA magnification related to OK treatment, which does not reflect on VEPs’ response.

In OK treatment, the myopic shift is expected to bring the hyperopic defocus to the retina to create an emmetropic or slightly myopic periphery. We hypothesized that this would possibly improve the retinal activity. However, not only did we not observe any significant improvement in PERG, but a delay from the P50 peak was also observed, as would be expected in cases of uncorrected defocus. However, in both PERG and VEPs methodologies, global retinal and cortical responses, respectively, are obtained. The lack of significant changes in both electrophysiological responses may be related to the fact that the changes in blur induced by OK treatment are not the same throughout the entire retinal eccentricity.

In the present study, we used the PERG methodology to evaluate possible changes in the electrical activity of the retina with the OK treatment. Although the technique is sensitive enough to changes in the innermost layers of the retina, and to the effect of blur and ametropia on the electrical activity, PERG response is mainly obtained by the sum of the total ganglion cells response, with different contributions of the macula and optic nerve. Thus, changes in more peripheral areas may not be observed with PERG. The multifocal ERG (mfERG) technique allows the obtaining of the electrical activity (driven primarily by photoreceptors and bipolar cells) in more localized/specific areas of the retina by allocating it into hexagonal blocks, using an m-sequence to stimulate each of these areas. Some studies comparing the retinal function of myopes with non-myopes found that the significant reductions on the amplitude and increased implicit times observed in myopic eyes are more pronounced at peripheral areas rather than the macula [17], even when considering the effect of the axial length [18,22]. In the case of OK treatment, the amount of blur induced by the rearrangement in the cornea structure is different from the center to the periphery [5,8,9], and so the changes induced in the periphery may be hidden from the overall response. In future studies, it would be useful to use multifocal techniques (mfERG and multifocal VEPs) to observe whether there are differences in response in different regions of the retina, and correlate it with the different zones of the CRT lens and the respective refractive changes in the peripheral retina, as well as with different levels of myopia.

## 5. Conclusions

In this study we found a slight increase in retinal and cortical response amplitude, observed with both PERG and VEP, respectively, and these differences were not statistically significant during the 60 days of OK treatment. This shows that retinal and cortical visual-related electrical activity is maintained or slightly increased during OK treatment.

## Figures and Tables

**Table 1:** Descriptive Statistics (Mean ± SD) for Population Data Collection of refractive error and visual acuity.

	M (D)	J0 (D)	J45 (D)	HCDVA ^1^	LCDVA ^2^
Pre-treatment	−1.60 ± 0.35	0.05 ± 0.22	−0.02 ± 0.21	−0.09 ± 0.08	0.08 ± 0.08
Post-treatment	−0.05 ± 0.36	−0.03 ± 0.15	0.01 ± 0.24	−0.11 ± 0.11	0.15 ± 0.13
*p*	<0.001 *	>0.050 ^+^	>0.050 ^+^	<0.050 ^+^	>0.050 ^+^

* Paired samples *t*-test ^+^ Wilcoxon test ^1^ High contrast logMar visual acuity. ^2^ Low contrast logMar visual acuity.

**Table 2:** Description (Mean ± SD) of the variation of the components of the PERG wave [implicit time of N35, P50 and N95 (expressed in ms) and the amplitude of P50 and N95 (expressed in μV)].

		Baseline	1 Night	15 Nights	30 Nights	60 Nights	*p*
Implicit time (ms)	N35	29.83 ± 3.72	29.18 ± 3.84	30.26 ± 2.07	30.03 ± 3.49	29.96 ± 2.56	0.649 ^+^
	P50	53.09 ± 3.45	51.13 ± 4.30	53.57 ± 2.78	52.69 ± 4.11	52.05 ± 2.86	0.032 ^+^
	N95	93.04 ± 6.53	97.05 ± 9.26	93.52 ± 6.97	96.88 ± 6.17	98.14 ± 5.30	(a) 0.008 ^+^
Amplitude (μV)	P50	3.68 ± 1.13	3.68 ± 1.11	4.09 ± 1.20	3.56 ± 0.90	3.70 ± 1.27	0.469 ^+^
	N95	5.09 ± 1.53	5.82 ± 1.93	6.26 ± 1.63	5.96 ± 1.64	6.04 ± 1.89	0.430 ^+^

(+) Friedman test. Statistically significant differences (*p*) among the visits highlighted. (a) Bonferroni post hoc test—statistically significant differences only for N95 implicit time between B−60N (visits Baseline and 60 nights) and 15N−60N (visits 15 nights and 60 nights).

**Table 3:** Description (Mean ± SD) of the variation of the components of the VEP wave (implicit time of N75 and P100 (expressed in ms) and the amplitude of P100 (expressed in μV)).

		Baseline	1 Night	15 Nights	30 Nights	60 Nights	*p* (a)
Implicit time (ms)	N75	68.41 ± 5.26	70.36 ± 4.21	70.57 ± 3.85	71.22 ± 5.44	70.61 ± 3.70	0.681 ^+^
	P100	101.60 ± 4.96	102.41 ± 5.04	102.60 ± 4.51	104.35 ± 4.40	102.66 ± 5.04	0.118 *
Amplitude (μV)	P100	15.45 ± 5.79	16.76 ± 6.46	15.68 ± 6.64	14.97 ± 6.11	16.90 ± 4.95	0.093 ^+^

(*) ANOVA repeated measures; (^+^) Friedman test.
